# Cone beam computed tomography diagnostic imaging of intra-osseous mucoepidermoid carcinoma in the mandible

**DOI:** 10.4317/jced.53785

**Published:** 2017-09-01

**Authors:** Andre-Luiz-Ferreira Costa, Thasia-Luiz-Dias Ferreira, Haroldo-Arid Soares, Ana-Carla-Raphaelli Nahas-Scocate, Gonzalo-André-Parra Montesinos, Paulo-Henrique Braz-Silva

**Affiliations:** 1 PhD,DDS, Department of Orthodontics and Radiology, University of City of Sao Paulo, Sao Paulo, SP, Brazil; 2 PhD, DDS, Regional Hospital Dr. Carmino Caricchio, Division of Stomatology, Sao Paulo, SP, Brazil; 3 MSc, Department of Orthodontics and Radiology, University of City of Sao Paulo, Sao Paulo, SP, Brazil; 4 PhD, DDS, Division of General Pathology, Department of Stomatology, School of Dentistry, University of Sao Paulo, Sao Paulo, SP, Brazil

## Abstract

Intra-osseous mucoepidermoid carcinoma in the mandible is a rarely reported entity, comprising only 2-3% of all mucoepidermoid carcinomas. Unilocular and/or multilocular radiolucency is characteristic of mucoepidermoid carcinoma, but has a radiographic appearance similar to that of odontogenic benign and malignant tumors and thus cannot be accurately diagnosed on plain films. This article describes a case of a 36-year-old man with intra-osseous mucoepidermoid carcinoma in the left mandible. For a detailed analysis, both panoramic radiograph and cone beam computed tomograph were taken. Important clinico-pathological and imaging features, differential diagnosis and review of the literature are described.

** Key words:**Diagnosis, Cone Beam Computed Tomography, Head and Neck Tumors.

## Introduction

Intra-osseous mucoepidermoid carcinoma (IMC) in the mandible is a rare bone malignant neoplasm, comprising 2-4% of all mucoepidermoid carcinomas diagnosed (1). For some authors, these lesions are classified more appropriately as odontogenic tumors rather than as salivary gland neoplasms (2). Imaging plays an important role in the detection and differentiation of IMC because its sclerotic periphery and mixed internal structure, consisting of unilocular and/or multilocular pattern with imaging characteristics similar to those of other lesions, including ameloblastoma, glandular odontogenic cyst and keratocystic odontogenic tumour (1). This case report intends to highlight the value of cone beam computed tomography (CBCT) for the diagnosis of a patient with IMC in mandible as it helps to delineate the lesion and plan the treatment properly.

## Case report

**Figure 1:**
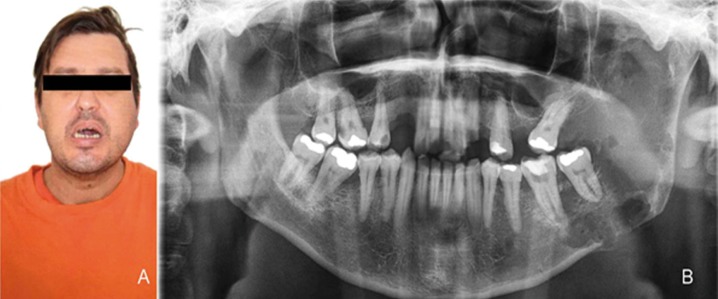
A: Extra-oral image of the affected area, with marked facial swelling on the left mandibular side and trismus; B: An irregular, ill-defined diffuse radiolucency located in the posterior region of the mandibular body, affecting teeth 37 and 36 and almost the whole left mandibular ramus with destruction of cortical basal bone.

**Figure 2:**
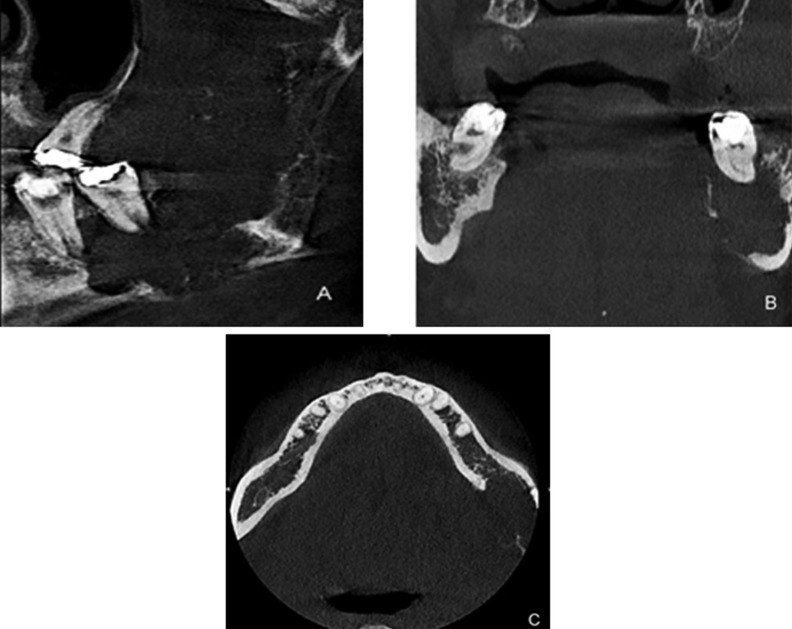
A: Sagittal CBCT image reveals expansile hypodense lesion in the inferior portion of the left ramus with thinning and expansion of the cortical portion; B: Coronal view an expansile lesion involving the left mandible: note the bone destruction; C: Image showing axial view.

A 37-year-old male patient presented to the Division of Stomatology of the Carmino Carrichio Regional Hospital, São Paulo, SP, Brazil, complaining of a painful left-cheek swelling for approximately three months. Trismus and paraesthesia were observed. The patient’s past medical history was unremarkable as he denied smoking and consuming alcohol. On extra-oral examination, a facial asymmetry with a smooth, hard and fixed swelling at the left mandible was noticed (Fig. 1A). Clinical intraoral examination showed a large ulcerated lesion with buccal and lingual bone expansion around the left body and ramus of the mandible. The associated teeth were sound, but with noticeable mobility. The panoramic radiograph revealed irregular unilocular radiolucency extending horizontally from posterior region of the body to the whole ramus and angle of the mandible (Fig. 1B). Erosion of the inferior border of the mandible with irregular, ill-defined areas of bone destruction was observed. The patient was also submitted to CBCT examination for better evaluation of both extension and form of the mandibular lesion. CBCT scan images (Kodak CS8100 3D system, Kodak Carestream Health, Paris, France) showed an expansive lesion in the left mandible with destruction of posterior body and extend into ramus. Irregular hypodensity was observed with diffuse limits, covering the posterior region of the body, angle and ramus as well as lateromedial expansion and bone erosion of the ramus of the mandible (sagittal view, Fig. 2A), compromising the alveolar supporting bone of teeth #36 and #37. Coronal CBCT view showed an expansile hypodense lesion, erosion of lingual cortical plate (Fig. 2B), whereas axial CBCT view (Fig. 2C) showed an ill-defined soft tissue density mass, and extension of the lesion within the mandible with break in lingual cortex

**Figure 3:**
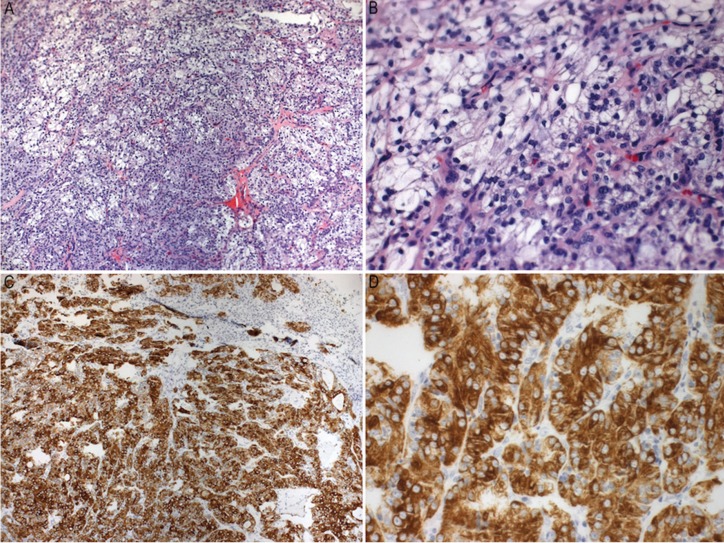
A,B: Histopathological features of intra-osseous mucoepidermoid carcinoma: Proliferation of epithelial, mucous and intermediate cells (H&E staining, original magnification A 100X, B 200X). C,D: CK-7 positive diffuse immunoexpression (streptavidin-biotin-peroxidase, original magnification C 100X, D 200X).

Based on clinical and radiographic aspects, the differential diagnosis included ameloblastic carcinoma and primary intra-osseous carcinoma. Under local anesthesia, incisional biopsy was performed. Histopathological sections revealed cystic and solid patterns, with proliferation of epidermoid, mucous and intermediate cells (Fig. 3A). Neither significant pleomorphism nor mitotic activity was observed (Fig. 3B). Cytokeratin 7 (CK-7) immunohistochemistry showed a diffuse expression, which confirms the glandular epithelial differentiation of the tumor (Fig. 3C,D). Based on the histopathological features, the final diagnosis was an intermediate-grade intra-osseous mucoepidermoid carcinoma.

The patient was referred to the State of São Paulo Cancer Institute (ICESP) for treatment, with complete resection of the lesion and adjuvant radiotherapy being performed. The patient has been followed up since then and feeling well.

## Discussion

The aetiology of IMC is still unclear (3). Some studies suggest three origins (3,4), namely: embryonic salivary gland remnants included within the mandible or mucous glands entrapped within the bone; transformation of mucous cells found in odontogenic cysts; and submucosal mucous glands with intra-osseous extension. The tumour is usually located in the mandible (premolar-molar-angle region), occurring three times more often in the maxilla (5). Also, it affects women twice more than men (5,6) and it has been reported to occur in all ages, most commonly in the 4th and 5th decades of life (3,5).

Reports of IMC originating from jaws are remarkably uncommon and often involve swelling and pain, whereas trismus, paraesthesia and tooth mobility are less frequently noticed (7). He *et al.* (3) reported a total of 24 cases and Bell *et al.* (5) reviewed 25 cases. Only one report focused on diagnostic imaging features (1). Radiographically, these lesions may present unilocular or multilocular radiolucency (7) with diffuse bone resorption and extensive loss of cortical bone (8).

Radiographic examination is important for categorization of a central jaw lesion (7,9). Brookstone and Huvos (10) proposed a three-grade classification for IMC.

- Grade 1: without expansion and rupture of cortical plate; - Grade 2: with expansion but without rupture of cortical plate; - Grade 3: with rupture of cortical plates or presence of regional metastasis.

The present case was classified as grade 3, that is, with no metastasis being detected. The radiographic features of IMC typically show marked variation in the appearance of the borders, and consequently it is worth regarding them as a differential diagnosis of jaw radiolucency (8,9). Panoramic radiography and conventional computed tomography (CT) are routinely used as diagnostic tools for evaluating the maxillofacial area.

However, panoramic radiography does not allow assessment of destructive bone lesions with non-uniform border or with degrees of extension and invasion into the surrounding tissues (8), nor enough to differentiate this kind of tumor from an odontogenic cyst or ameloblastoma. On the other hand, CT offers a large amount of information on size, location and area of the tumor in the region (11).

In 2013, Chan *et al.* (1) analysed the radiographic features of four cases using CT imaging. The internal pattern comprises disseminated foci of amorphous sclerotic bone, which appear as irregular masses with cortical bone-like structure without any organization into trabeculae or septa. Multiple small radiolucent loculations measuring less than 8 mm with and without rough borders in the septum were also observed.

This case report describes the value of using CBCT in the diagnoses of IMC. CBCT provides images of the lesion in all planes and thus enables localization, extension and internal structure of it. In fact, CBCT confirmed the sclerotic periphery and mixed internal structure of the lesion by highlighting border expansion and thinning of the cortical plate with erosion through the cortex.

These lesions are treated with radical resection of the margin of the adjacent normal bone. Neck dissection and post-operative radiation therapy may be required to control spread to lymph nodes (2). The lesion in our case was treated with radical resection and adjuvant radiotherapy.

To the best of our knowledge, the present case report illustrates the first use of CBCT for diagnostic approach of this rare malig-nancy.

## References

[1] Chan KC, Pharoah M, Lee L, Weinreb I, Perez-Ordonez B (2013). Intraosseous mucoepidermoid carcinoma: a review of the diagnostic imaging features of four jaw cases. Dentomaxillofac Radiol.

[2] Pires FR, Paes de Almeida O, Lopes MA, Elias da Cruz Perez D, Kowalski LP (2003). Central mucoepidermoid carcinoma of the mandible: report of four cases with long-term follow-up. Int J Oral Maxillofac Surg.

[3] He Y, Wang J, Fu HH, Zhang ZY, Zhuang QW (2012). Intraosseous mucoepidermoid carcinoma of jaws: report of 24 cases. Oral Surg Oral Med Oral Pathol Oral Radiol.

[4] Johnson B, Velez I (2008). Central mucoepidermoid carcinoma with an atypical radiographic appearance. Oral Surg Oral Med Oral Pathol Oral Radiol Endod.

[5] Bell D, Lewis C, El-Naggar AK, Weber RS (2016). Primary intraosseous mucoepidermoid carcinoma of the jaw: Reappraisal of The MD Anderson Cancer Center experience. Head Neck.

[6] Eversole LR, Sabes WR, Rovin S (1975). Aggressive growth and neoplastic potential of odontogenic cysts: with special reference to central epidermoid and mucoepidermoid carcinomas. Cancer.

[7] Raut D, Khedkar S (2009). Primary intraosseous mucoepidermoid carcinoma of the maxilla: a case report and review of literature. Dentomaxillofac Radiol.

[8] Adachi M, Inagaki T, Ehara Y, Azuma M, Kurenuma A, Motohashi M (2014). Primary intraosseous carcinoma arising from an odontogenic cyst: A case report. Oncol Lett.

[9] Kaffe I, Ardekian L, Peled M, Machtey E, Laufer D (1998). Radiological features of primary intra-osseous carcinoma of the jaws. Analysis of the literature and report of a new case. Dentomaxillofac Radiol.

[10] Brookstone MS, Huvos AG (1992). Central salivary gland tumors of the maxilla and mandible: a clinicopathologic study of 11 cases with an analysis of the literature. J Oral Maxillofac Surg.

[11] Sukovic P (2003;6 Suppl 1:31-6). Cone beam computed tomography in craniofacial imaging. Orthod Craniofac Res.

